# Transforming Growth Factor-Beta and Urokinase-Type Plasminogen Activator: Dangerous Partners in Tumorigenesis—Implications in Skin Cancer

**DOI:** 10.1155/2013/597927

**Published:** 2013-07-18

**Authors:** Juan F. Santibanez

**Affiliations:** Laboratory for Experimental Hematology and Stem Cells, Institute for Medical Research, University of Belgrade, Dr. Subotića 4, 11129 Belgrade, Serbia

## Abstract

Transforming growth factor-beta (TGF-**β**) is a pleiotropic factor, with several different roles in health and disease. TGF-**β** has been postulated as a dual factor in tumor progression, since it represses epithelial tumor development in early stages, whereas it stimulates tumor progression in advanced stages. During tumorigenesis, cancer cells acquire the capacity to migrate and invade surrounding tissues and to metastasize different organs. The urokinase-type plasminogen activator (uPA) system, comprising uPA, the uPA cell surface receptor, and plasminogen-plasmin, is involved in the proteolytic degradation of the extracellular matrix and regulates key cellular events by activating intracellular signal pathways, which together allow cancer cells to survive, thus, enhancing cell malignance during tumor progression. Due to their importance, uPA and its receptor are tightly transcriptionally regulated in normal development, but are deregulated in cancer, when their activity and expression are related to further development of cancer. TGF-**β** regulates uPA expression in cancer cells, while uPA, by plasminogen activation, may activate the secreted latent TGF-**β**, thus, producing a pernicious cycle which contributes to the enhancement of tumor progression. Here we review the specific roles and the interplay between TGF-**β** and uPA system in cancer cells and their implication in skin cancer.

## 1. Introduction

Metastasis results from a complex molecular cascade which allows cancer cells to leave the site of the primary tumor mass and to disseminate to distant anatomical sites where they proliferate and form secondary tumour foci. Disseminated disease is the most usual cause of death in cancer patients and is, therefore, a very serious clinical problem [1].

Transforming growth factor-beta (TGF-*β*) has been postulated to have a dual role in tumour progression, acting as a tumour suppressor in early stages of carcinogenesis, and exerting a prooncogenic role in the last steps of the metastatic disease [2]. TGF-*β* induces the epithelial mesenchymal transition (EMT) of transformed cells, which contributes to tumour invasion and metastasis, and is frequently overexpressed in carcinoma cells [3–7].

To invade and metastasize, cancer cells traverse the surrounding extracellular matrix (ECM) expressing a set of ECM degrading proteases, such as urokinase-type plasminogen activator (uPA), which plays a key role in cells' invasion and metastasis. uPA converts plasminogen to plasmin, which in turn can degrade a wide variety of ECM components and enable the tumour cells to penetrate the basement membrane [8, 9]. In addition, uPA, by binding to its cell surface receptor (uPAR), also modulates cell adhesion, proliferation, and migration [10, 11]. Consistent with its role in cancer dissemination, the high level of uPA correlates with the adverse patient outcome [12, 13].

The aim of this review paper is to reflect on TGF-*β* as key molecule in cancer and its molecular interplay with the uPA system, taking into account that both are involved in the complex cascade of events that culminate in cancer cell metastasis with possible implications in skin cancer.

## 2. Transforming Growth Factor-Beta

### 2.1. Signaling Pathways Initiated by TGF-*β*

The TGF-*β* superfamily of secreted growth factors comprises more than 40 ligands that, despite exhibiting pronounced structural similarities (such as their dimeric structure and presence of a cysteine knot motif), function as regulators of a variety of divergent processes both during embryogenesis and later on in adult homeostasis and also participate in tumorigenesis [14, 15].

Transforming growth factors were discovered in studies of platelet-derived growth factor (PDGF) and epidermal growth factors (EGF/TGF*α*) and were named according to their capacity to “transform” fibroblast rat cells *in vitro* [16]. Six distinct isoforms of TGF-*β* with a degree of homology of 64–82% have been discovered, although only the TGF-*β*1, -*β*2, and -*β*3 isoforms are expressed in mammals [17]. The expression of the three isoforms is differently regulated at the transcriptional level due to different promoter sequences. TGF-*β*1 promoter lacks the classic TATAA box but possesses multiple regulatory sites that can be activated by a number of immediate early genes and oncogenes and is inhibited by tumor suppressors [18]. The TGF-*β*2 and -*β*3 promoters each contain TATAA boxes and a common proximal CRE-ATF site, suggesting their role in hormonal and developmental control [19].

### 2.2. The TGF-*β* Receptor Family

**Figure 1:**
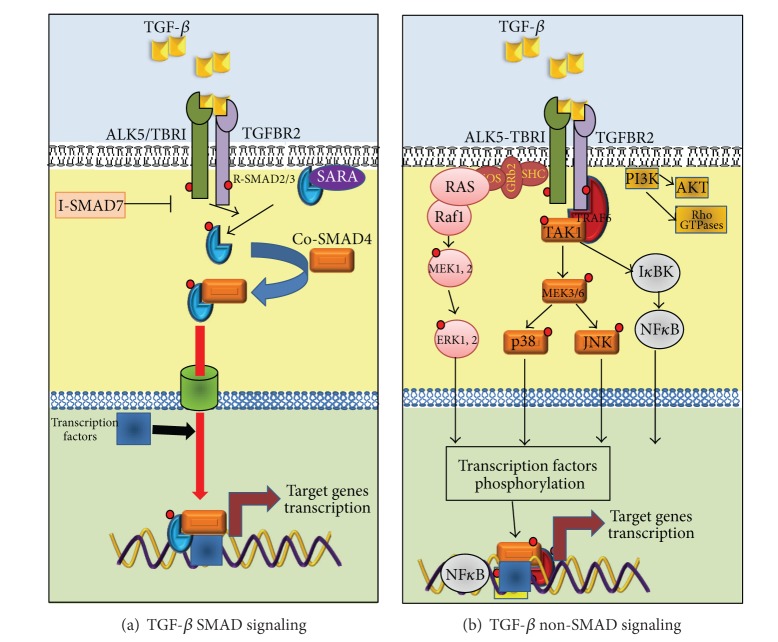
TGF-*β* signaling. TGF-*β* signaling comprises two groups of a set of intracellular transduction pathway: SMADs signals and Non-SMADs signals. When the active TGF-*β*1 binds to its cell surface type II receptor (TBR2), it induces the activation of TGF-*β* type I receptor (ALK5 or TBRI) and forms a heterotetrameric complex. (a) SMADs signals: active ALK5 in the complex phosphorylates SMAD2/3 which in turn promotes the SMADs release from complexes with SARA from the inner face of the plasmatic membrane. Phosphorylated SMADs interact with co-SMAD4 forming a heteromeric complex to be translocated into the cell nucleus, where, by interacting with other transcription factors and/or co-repressors or co-activators, they modulate gene expression. (b) Non-SMAD signals: active TGF-*β* receptors complex interacts with ubiquitin ligase tumor necrosis factor receptor-associated factor 6 (TRAF6) which in turn recruits TGF-*β*-activated kinase 1 (TAK1) to activate p38, JNK, and NF*κ*B pathways. Additionally, TGF-*β* binding provokes the phosphorylation of ALK at tyrosine residues which enable the formation of Shc-Grb2/SoS complex to activate Ras-Raf1-MEK1,2-ERK1,2 signaling. On the other hand, receptor-activated complexes can activate PI3K provoking the activation of AKT and the small Rho GTPases. The activation of Non-SMAD signals pathways in turn initiates transcriptional or nontranscriptional activities to regulate cellular responses.

TGF-*β* family members bind to their cell surface receptors to form heteromeric complexes. Dimers of type I and type II serine/threonine kinase receptors interact with the dimeric ligands (Figure 1). Seven type I (ALK1–7) and five type II receptors (TGFBR2, BMPR2, ACVR2, ACVR2B, and AMHR2) have been described. Differential affinities for the individual ligand contribute to signaling specificity, that is, TGF-*β* binds specifically to ALK5 or TBRI and TGFBR2 [14]. In addition, TGF-*β* ligands can interact with the coreceptors, type III receptors, and endoglin and betaglycan, which both drive ligand binding and modulate the receptor kinase transduction [20].

TGF-*β* receptors are subject to posttranslational modifications, such as phosphorylation/dephosphorylation, sumoylation, and ubiquitylation, which regulate their stability and availability. These modifications are part of the fine tuning involved in the TGF-*β* superfamily signal transduction modulation, resulting as key determinants in the TGF-*β* cellular responses [15].

Another point of modulation is the regulation of the level of TGF-*β* receptors. The ligand/receptor complexes can be internalized via lipid rafts/caveolae to be degraded inside a proteasome [21]. The TGF-*β* receptor degradation is dependent on its association with Inhibitory SMADs (SMAD6 and SMAD7) and HECT type E3 ligases SMURF1 and SMURF2 (SMURF ubiquitin ligases). Thus, SMURFs/I-SMADs regulate the cellular pool of TGF-*β* receptors and inhibit TGF-*β* superfamily signaling. SMAD6 and SMAD7 recruit SMURF ubiquitin ligases to induce ubiquitination and degradation of TGF-*β* receptors [22].

After binding to the type I and type II serine/threonine kinase receptors (TBRI and TGFBR2, resp.), TGF-*β* causes their hetero-oligomerization which subsequently activates different intracellular signaling pathways. TBRI is phosphorylated at the “GS” domain by the constitutively active receptor type II producing a ligand-receptor complex in an activated state [23]. In addition, the phosphorylation of the GS domain changes it to more acidic surface ambient allowing the recruitment of the downstream effectors SMADs which are then phosphorylated by receptor type I through the interaction with the SMADs' basic domains [24].

### 2.3. SMAD-Dependent Signaling Initiated by TGF-*β*

The activated receptor complexes transduce intracellular signaling by the type I receptor phosphorylation of SMAD proteins in their carboxy-terminal domains. In unphosphorylated form, the SMADs are transcriptionally inactive and sequestered by the cytoplasmic retention proteins such as SARA (SMAD anchor for receptor activation) [25].

TGF-*β* receptors phosphorylate SMAD2 and SMAD3, also classified as receptor associated-SMADs (R-SMADs) [14] (Figure 1(a)). R-SMAD proteins consist of three domains: two highly conserved domains at the N-terminus and the MH1 (MAD homologous region 1) domain at the C-terminus of the protein which can interact with other proteins and possesses a nuclear localization signal (NLS); or MH2 (MAD homologous region 2) domain that mediates homo- or hetero-oligomerization of the SMADs and the transactivation of SMAD nuclear complexes, respectively. A highly variable linker region exists between MH1 and MH2 domains; it is enriched in prolines and is a potential serine/threonine substrate for phosphorylation [25].

All activated R-SMADs, after being phosphorylated by the TGF-*β* receptors, are released from the cytoplasmic membrane and interact with the common SMAD (SMAD4 or co-SMAD). SMAD4 has an insertion in the MH2 motif and lacks the C-terminal motif for type I receptor phosphorylation. The activated SMADs complex, a trimer consisting of a single co-SMAD and homo- or hetero-dimer of R-SMADs, is then shuttled into the nucleus where it binds to promoters of the target genes with other transcription factors [26]. Two of these genes are the third component of the SMADs family, the Inhibitory SMADs (I-SMADs): SMAD6 and SMAD7. I-SMADs' expression produces a negative-feedback regulation of TGF-*β* signaling. I-SMAD proteins contain a characteristic C-terminal MH2 domain, but they lack the conserved MH1 domain. SMAD7 inhibits R-SMAD phosphorylation by binding the TGF-*β* receptors, while SMAD6 preferentially inhibits BMP signaling [27].

In the nucleus, SMAD proteins complexes can bind directly to DNA with weak affinity to SMAD-binding elements (SBEs) to regulate the transcription of target genes. SMAD3/SMAD4 complexes recognize a 5-base pair, GTCTG or CAGAC [28].

In the SMAD2 protein, a 30-amino-acid insertion encoded by exon 3 in the MH1 domain disables its binding to DNA. The binding of SMAD complexes to DNA, although at a low affinity, has been shown to be crucial for the transcriptional activation of SMADs' target genes, and certainly the binding to the chromatin requires interactions with transcription factors to form transcriptional complex with high affinity to DNA [26].

### 2.4. Non-SMAD Signaling Pathways Initiated by TGF-*β*

The relative simplicity of the SMAD signaling model produces a dilemma in terms of understanding the plethoric diversity of functions of the TGF-*β*. Is it well known that the TGF-*β* superfamily signaling is not limited to SMAD-mediated pathways, but is determined by a crosstalk of non-SMAD pathway components which may in an alternate way modulate cellular responses [15]. These non-SMAD pathways include mitogen-activated protein kinase (MAPK) pathways, NF-*kB* pathway, Rho-like GTPase signaling pathways, and phosphatidylinositol-3-kinase (PI3K)/AKT pathways (Figure 1(b)) [14]. Briefly, one of the first indications that TGF-*β* activates a pathway different than SMADs came from the observation of Ras activation by TGF-*β* in epithelial cells [29], allowing the possibility that TGF-*β* may also activate ERKs MAPK. Recently, Lee et al. [30] demonstrated that the type I TGF-*β* receptor ALK5 can, after being tyrosine phosphorylated by TGF-*β*, recruit and phosphorylate both serine and tyrosine residues in the ShcA adaptor, thus, promoting the formation of a ShcA/Grb2/Sos complex. This triggers the activation of RAS-RAF-ERK MAPK cascade which can regulate cell growth, proliferation, or migration.

TGF-*β*, independent of receptor's kinase activity, is also able to activate the p38 and c-Jun N-terminal kinase (JNK) MAPKs, by the recruitment of the ubiquitin ligase tumor necrosis factor receptor-associated factor 6 (TRAF6) to the ALK5 cytoplasmic domain, which in turn activates TAK1, MEKK4, and MEKK3/6 to produce the activation of JNK and p38, respectively, further regulating apoptosis, differentiation, or cell migration [31, 32].

Like MAPK pathways, the Rho-like GTPases, including RhoA, Rac, and Cdc42 are also key players in TGF-*β* signaling. TGFBR2 phosphorylates the polarity protein PAR6, which regulates the local degradation of RhoA, which in turn produces a tight junctions disassembly and a rearrangement of actin cytoskeleton. This epithelial architecture disintegration as a consequence induces the epithelial to mesenchymal transition (EMT), an important developmental and disease-associated process that is regulated by TGF-*β* signaling [33]. Finally, and similarly to various growth factors, TGF-*β* has been shown to rapidly activate PI3 kinase, leading to the activation of the Akt kinase, in diverse cell systems. This activation appears to be independent of SMAD2/3 activation, whereas the kinase activities of the TGF-*β* receptors are required for TGF-*β*-induced PI3K activation. Interestingly, the PI3K/Akt pathway may antagonize SMAD-mediated effects and protect cells from TGF-*β*-induced apoptosis and growth inhibition [14, 34].

## 3. The Urokinase-Type Plasminogen Activator System

The urokinase-type plasminogen activator system consists of uPA, the uPA receptor (uPAR), the substrate plasminogen (Plg), the plasminogen activator inhibitor 1 (PAI1; also known as SERPINE1), and PAI2 (also known as SERPINE2). uPA system has attracted attention for its wide range of targets as well as its prominent location in the proteolytic network of tumors [35].

### 3.1. uPA, uPAR, and Plg

**Figure 2:**
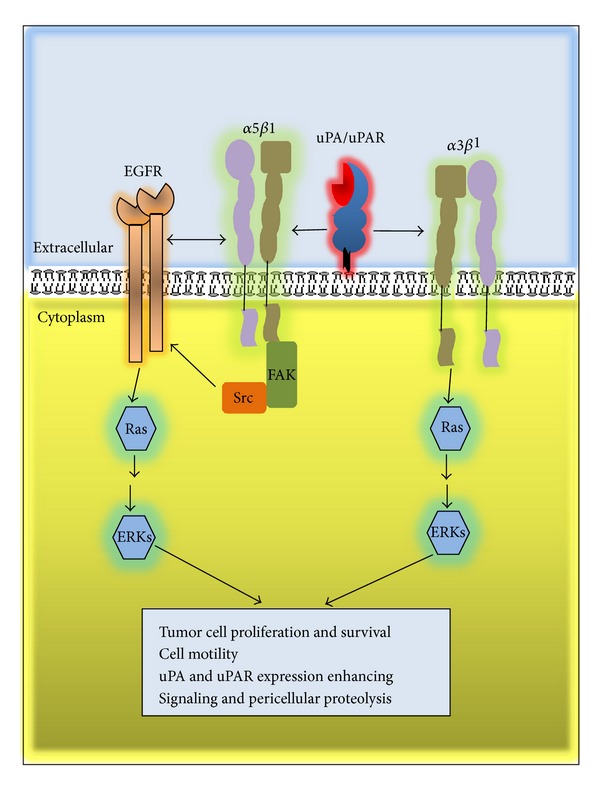
uPA/uPAR signal transduction. The binding of uPA to uPAR can trigger the activation of intracellular signal transduction either by interacting with *α*5*β*1 integrin which allows the transactivation of EGFR by a mechanism mediated by FAK and Scr to in turn activate RAS-ERK1 MAPK, or by interacting with *α*6*β*1 integrin to also activate RAS-ERK signal.

uPA is best known for its ability to convert plasminogen into plasmin (Figure 2) [36]. It is synthesized as a nonactive single chain (sc-uPA) 54-kd glycoprotein containing 411 amino acids. uPA protein can be divided into three functionally independent regions: the amino terminal domain containing an epidermal growth factor- (EGF-) like domain/growth factor domain (GFD) (residues 5–49) with the capacity to bind to uPAR with high affinity (Kd ∗ 10^−10^ − 10^−9^ mol/L) [37, 38]; the kringle domain (residues 50–131), implicated in intracellular signaling and in the induction of cell migration and adhesion [39]; the carboxy-terminal catalytic domain which in excess of plasmin (Plm) can be released by hydrolysis of the Lys135-Lys136 peptide bond after previous cleavage of the Lys158-Ile159 bond to generate a low molecular weight two-chain uPA (33 kd, tc-uPA). uPA lacking the GFD and unable to interact with uPAR undergoes rapid endocytosis and intracellular degradation [39]. The first two domains comprise the amino-terminal fragment (ATF) [35, 40].

Binding of sc-uPA to uPAR on the cell surface is crucial for its activation under physiological conditions. uPAR is a heterogeneously glycosylated protein of 50 to 60 kDa, synthesized as a 313-amino-acid polypeptide, anchored to the plasma membrane by a glycosylphosphatidylinositol moiety. The uPAR molecule is composed of three related structural domains (D1, D2, and D3), all three involved in a combined binding site in the central cavity of the receptor to generate high-affinity binding of uPA via its GFD [41–43]. Alternatively, uPAR can protect the bound uPA from further degradation by plasmin [39].

Human plasminogen is a 92 kDa, single-chain glycoprotein consisting of 791 amino acids; it contains 24 disulfide bridges and five homologous kringles. uPA converts plasminogen to plasmin by cleavage of a single Arg561-Val562 peptide bond [42]. Plasminogen, similarly to uPA, can bind to specific cell surface receptors to form a highly localized point of proteolysis [35]. The binding of sc-uPA to uPAR strongly enhances Plg cleavage to generate active plasmin. Further on, a positive feedback is produced since plasmin, by a proteolytic cleavage of the Lys158-Ile159 peptide bond, converts latent sc-uPA to an active two-chain uPA (tc-uPA). Moreover, a feedback loop is also created by uPA and plasmin which can activate each other. Also, cathepsin-B or kallikreins 2, 4, and 12 can activate pro-uPA [44]. Additionally, cell-associated plasmin, bound to S100A10 (a highly inducible plasminogen receptor), is protected from rapid inhibition by *α*2-antiplasmin, which further favors the activation of receptor-bound sc-uPA and also serves to the proteolytic activity of focalized plasmin [35, 42, 45, 46]. The uPA activation system is negatively regulated by PAI1 and PAI2 which can covalently bind to their targets to inhibit proteolytic activity [47]. Furthermore, thrombin hydrolysis provides the mechanism of proteolytic inactivation of uPA cleavage of the Arg156-Phe157 enzyme bond that does not exclude nonproteolytic functioning of such peptide forms [35].

Plasmin cleaves range ECM components and is essential for the degradation and clearance of fibrin blood clots (fibrinolysis) during wound healing. Plasmin can also activate matrix metalloproteinases (MMPs), such as MMP2, MMP3, MMP9, MMP12, and MMP13 [35, 48–50]. Accelerated cell-associated plasminogen activation by uPA/uPAR can facilitate cell migration through a three-dimensional ECM by enhancing pericellular proteolysis. Localization of uPAR to the leading edge of migrating cells exerts spatial control over ECM degradation by focusing uPA activity on the direction of the movement [51]. Importantly, plasmin and MMPs can also release ECM-bound growth factors or activate latent growth factors including TGF-*β*1, as mentioned above [40, 52, 53].

In migrating cells, the coordinated expression of uPA and uPAR exists at cell-substrate and cell-cell contact sites [35, 40]. uPA/uPAR complexes focalize plasmin production to initiate extracellular matrix proteolysis, at the same time disrupting cell-cell contact and increasing cell motility. Plasmin inhibitors can suppress cell migration both *in vitro* [54, 55] and *in vivo* [56, 57], suggesting an important role of plasmin-induced proteolysis in this process. Urokinase proteolytically modifies the ECM environment and affects matrix proteins that are the ligands of the integrin receptors associated with the intracellular signaling systems, thus, regulating cytoskeleton rearrangements, adhesive contacts, and chemotaxis [58].

### 3.2. uPA/uPAR Signaling

Numerous studies indicate that the uPA/uPAR complex has different roles beyond the regulation of extracellular proteolysis. Binding of uPA to uPAR triggers the activation of intracellular signals that promote migration, invasion, adhesion, differentiation, proliferation, and cell survival [8–10, 35]. The initiation of signal transduction depends upon its association with transmembrane proteins, including members of the integrin family [59], chemotactic receptors [60], and receptor tyrosine kinases, such as the EGFR [61–63]. Although the association of uPAR with these proteins is well documented, the underlying molecular mechanisms and factors that modulate the uPAR signaling response are not well understood. 

Signaling through uPAR activates the Ras-MAPK pathway, p38, focal adhesion kinase (FAK), Src, and the Rho family small GTPase Rac1 [64–67]. Additionally, uPA/uPAR can activate JAK1-STAT1 and PI3K pathways [35, 40, 68].

Although the expression of uPAR and its ability to bind uPA are required for signaling, it is independent of the proteolytic activity of uPA. Chemically inactive uPA or nonproteolytic uPA derivates (such as sc-uPA or ATF) effectively activate intracellular signaling [40, 60, 69].

Ligands other than uPA, that bind to uPAR, such as the ECM glycoprotein vitronectin, usually bind at the outer side of the receptor, and because binding sites are different, uPAR can simultaneously bind both ligands and activate downstream signaling [70–72]. In addition, uPA/uPAR complex can indirectly bind to vitronectin through PAI1 [71].

Since uPAR lacks transmembrane and intracellular domains, the cooperation with other transmembrane receptor is necessary to activate downstream signaling pathways, and one of the best known cooperating receptors is integrin, a major family of ECM receptors, including *α*5*β*1, *α*3*β*1, and *α*v*β*3 integrins (Figure 2) [71–75].

### 3.3. Endocytosis and Recycling of uPA/uPAR

**Figure 3:**
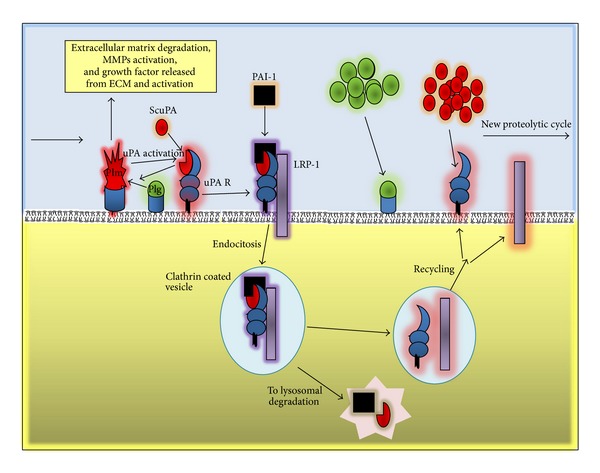
Regulation of uPA activity and uPAR recycling. ScuPA binds to its cell surface receptor uPAR and can be activated by Plm; activated uPA in turn is inhibited by PAI1 forming a complex with LPR1 which triggers the relocalization of uPA/uPAR into clathrin coated pits. In that moment, the activation of plasmin and degradation and invasion of extracellular matrix are reduced. The quaternary complex is internalized and uPA and PAI-1 are subjected to lysosomal degradation, while uPAR and LRP1 are recycled to new places in the plasmatic membrane, which could promote further new cycle of pericellular proteolysis enhancing extracellular matrix degradation, cell invasion, and metastasis.

One crucial step for the high effectiveness of pericellular proteolysis and cell invasion is the possibility of glycosylphosphatidylinositol (GPI) anchored uPAR regulation by endocytosis and further recycling to cell surface [35]. During the inhibition of uPA bound to uPAR by PAI1, when an inactive complex is formed in association with low density lipoprotein receptor-related protein-1 (LRP1 or a2 macroglobulin receptor), a clathrin-dependent endocytosis is triggered [76, 77]. This is when uPA and PAI1 are subjected to lysosomal degradation where uPA and LPR1 are being recycled to the plasma (Figure 3) [77].

The capacity of uPAR to be recycled to the cell membrane has a pivotal role in uPA/uPAR effects on cell migration. Endocytosis of uPA/uPAR/PAI1 may control the focalized pericellular proteolysis production and stop the ECM degradation concomitantly with changes in cell adhesion to the ECM, thus, enhancing cell migration. uPAR, through its localization in nascent integrins-containing adhesion complexes, activates intracellular signals transduction in cooperation with integrins and other transmembrane partners. Ligand-activated uPAR influences integrin-dependent cell adhesion, and acts as a nonintegrin vitronectin receptor [35, 40, 78]. The uPAR recycling provides a new focus for pericellular proteolysis, uPAR in association with endocytic receptor 180 (ENDO180 or uPAR-associated protein), a constitutively recycling collagen receptor of the mannose receptor family [79]. This interaction provokes an activation of Rho GTPases, Rac1, and Cdc42, which in turn induce the reorganization of actin cytoskeleton and direct cell migration towards the chemotactic gradient of uPA, producing a new focalized pericellular proteolysis and new ECM adhesions [77, 80].

Because of the GPI anchorage, the uPAR has high mobility in the plasma membrane, and its location depends on the functional state of the cell; whether the cell is resting or migrating, clusters of uPARs form on the leading edge [58]. The concentration of the proteolytic potential provides the vector movement of the cell along the chemoattractant gradient.

Additionally, endocytosis can temporarily reduce the amount of cell surface uPAR available for signaling, thereby, in a short time, preventing uPAR-mediated Rac1 and ERK1, 2 activation, inhibiting cell migration, and chemotaxis [81, 82], which may allow cells to accommodate to the new scenario of previous proteolytic modification of ECM. The promigrational effect caused by uPA/uPAR endocytosis ensuring the uPAR is recovered on the leading edge accelerates a new cycle of adhesion and cytoskeleton reorganization, which are required for cell movement along the substrate [83]. Thus, pericellular proteolysis, cell adhesion, migration, and invasion of tumor cells are a complex, finely tuned mechanism driven by uPA/uPAR, which converts this complex to a therapeutic target in tumor metastasis.

### 3.4. Plasminogen Receptors

When plasminogen binds to cells, its activation is markedly enhanced, compared to the reaction in the solution phase [84], and, as mentioned above, active plasmin associated with the cell surface is protected from inhibitors. Localization of plasminogen on cell surfaces is a crucial control point for positive regulation of the plasmin's proteolytic activity that facilitates both physiological and pathological processes [84, 85].

Described cell surface binding sites for plasminogen include *α*-enolase, annexin A2, p11, histone H2B, actin, gp330, cytokeratin 8, histidine-proline rich glycoprotein, and Plg-RTK [84, 85]. *α*-Enolase and most of these proteins exposing C-terminal lysine rich basic residues on cell surface are predominantly responsible for the ability of eukaryotic cells to enhance plasminogen activation [85]. Notably, most of these proteins have described functions other than plasminogen receptors and lack a transmembrane domain, Plg-RTK being an exception, as it is a transmembrane receptor [84]. Many of the characterized Plg receptors have a Kd of about 1 mM, and considering that plasma Plg concentration is about 2 mM, more than 50% of the receptors are binding Plg [86].

Additionally, beyond its role in the proteolytic activity on the cell surface, several recent studies have shown that plasmin is also able to activate several intracellular signaling pathways, which lead to the activation of several transcription factors, in a cell surface-binding-dependent way. At the moment, the underlying mechanisms are unknown, although it could operate by a direct binding of plasminogen/plasmin to its specific cell surface receptor or indirectly by proteolytic activation of growth factors [85]. The binding of Plg/Plm to cell surface receptors induces the activation of ERK, p38, and Janus kinase 1 (JAK1) that in turn activate AP-1 and NFkB [87, 88].

Although in most of the cases the receptor responsible for this cellular response remains to be identified [85], it is clear that the capacity and complexity of the formation of proteolytic cell surface complexes highly increases the effectiveness of ECM degradation and consequently facilitates cell migration.

## 4. Regulation of uPA, uPAR, and Plasminogen Expression

As we mentioned above, uPA system has been shown to play a key role in cell migration and tissue invasion by regulating both cell-associated proteolysis and cell-cell and cell-ECM. The expression and activity of the components of this complex system are strictly regulated. The control of the expression occurs both at transcriptional and posttranscriptional levels [89]. We will further focus on the transcriptional regulation of the uPA gene regulation as well as its modulation by TGF-b signaling.

### 4.1. Regulation of the uPA Gene Expression

The gene for uPA has been isolated from several mammalian species; the human uPA is encoded by the PLAU gene (Gene ID 5328), located at 10q24, which is 6.4 kb long and is organized in 11 exons and 10 introns. The uPA mRNA is 2.4 kb long with 900 bp of 3′-UTR conserved in several mammalian species [90–93]. Gene transcription is modulated by several regulatory elements identified in the 5′ flanking region [86, 94].

The 5′-flanking sequence contains several features that indicate tight transcriptional regulation [94]. At the upstream of the TATA box lies a GC-rich sequence of about 200 bases, variable in length depending on the species, containing several canonical and noncanonical binding sites for the ubiquitous transcription factor Sp1. The Sp1 has a prominent role in the constitutive expression of PLAU gene in cancer cells, and its DNA binding and transcriptional activity are modulated by a number of growth factors and signal transduction pathways [93, 95, 96].

There are two relevant regulatory regions highly conserved in human, mouse, and porcine uPA gene. The first regulatory region is an inducible enhancer located at 2 kb upstream of the transcription start site, which contains an Ets (PEA3)/AP-1 juxtaposed site followed by a noncanonical AP-1 about 70 bp downstream; this separating region is termed cooperation mediator (COM), which contains binding sequences for different uPA enhancer proteins [93, 97, 98]. The second is an Ets/AP-1 composite located at −536 bp in the opposite orientation [86].

Two additional regulatory elements have been found in the human promoter: an NF-kB binding site located at −1583 bp, which mediates the transcriptional induction of gene expression by phorbol esters in the absence of the enhancer AP-1 sites [99, 100]; two TBEs (T-cell factor binding elements) localized at the positions −737 bp and −562, which are bound by a transcription factor complex [86].

Interestingly, a SMAD binding element (SBE, CAGAC) is located in uPA promoter at about −175 bp without a specific role known until now. In addition, uPA promoter possesses a binding sequence for the transcription factor E2F (at −145 bp) which may play a role in uPA expression during cell proliferation [101, 102]. Intriguingly, TGF-*β* inhibits E2F1 transcription factor concomitantly with the inhibition of the proliferation of transformed epithelial cells, and active E2F1 strongly inhibits uPA expression [103]. In transformed epithelial cells, a mechanism by which TGF-*β*-inhibited E2F1 collaborates to increase uPA expression might operate; however, this mechanism remains to be elucidated.

### 4.2. Regulation of uPAR Expression

The human uPAR is encoded by the PLAUR gene located at 19q13 consisting of 7 exons (GeneID 5329) [86]. Under normal conditions, uPAR is thought to have fairly limited tissue expression. However in keratinocytes during epidermal wound healing, stress, injury, and inflammation can induce uPAR expression [35].

Several signaling pathways activate transcription factors that act on the uPAR promoter, driving uPAR expression in cancer [35, 104]. The human uPAR promoter was first described in 1994 [105]. *In vitro* studies have located the transcription start site 52 bp upstream to the translation start site (ATG). Similarly to housekeeping genes or constitutively expressed genes, it lacks TATA and CAAT boxes [106], close to the start site which contains a GC-rich proximal sequence with Sp1 consensus elements at −93 and −104 that regulate the basal expression of the gene.

In colon cancer, constitutive and PMA-inducible expression of the gene requires AP-1 consensus motif at −190/−171, which binds Jun-D, c-Jun, c-Fos, and Fra-1 transcription factors and mediates the transactivation of uPAR promoter through ERK and JNK MAPK pathways [107, 108].

Further studies demonstrated another region (−152/−135) of the uPAR promoter containing putative binding sites for (mismatched) Sp1, AP-2, and PEA3 binding motifs. These motifs are bound by an AP-2*α*-like protein being closely related to, however, not identical with, authentic AP-2*α*, Sp1 and Sp3 transcription factors. Binding of the AP-2*α*-like protein was found to be important for a constitutively high uPAR promoter activity in a highly invasive colon cancer cell line, and for PMA-stimulated uPAR expression in a cell line with low constitutive uPAR expression. Therefore, the two promoter elements, −190/−171 (AP-1) and −152/−135 (AP-2/Sp1/Sp3), appear as two key cis-elements regulating diverse means of uPAR control [104].

Several transcription factors have been implicated in the regulation of uPAR. Tumour hypoxia acts through hypoxia-inducible factor 1*α* (HIF1A) to drive uPAR expression through a hypoxia responsive element (HRE) in the uPAR promoter [109]. Nuclear factor-*κ*B (NF-*κ*B) also activates uPAR expression, either indirectly through HIF1A [110] or directly through a nonconsensus NF-*κ*B-binding site (−51/−30) in the uPAR promoter [111]. In addition, KLF4 bound to multiple sites of the proximal 200 bp of the uPAR promoter [112] and transcription factors of the T cell factor (TCF) and lymphoid enhancer-binding factor (LEF) protein family link uPAR expression to the activity of the Wnt pathway [113, 114].

uPAR gene is also subjected to negative regulation, for example, PEA3 bound to a PEA3/ets motif at −248 bp, via b3-integrin, acts as a transcriptional repressor; or by Sp3, which, by binding at −152/−135 bp, mediates the inhibition of the uPAR gene transcription by Programmed cell death protein-4 (Pdcd4) [104]. Thus, multiple signaling pathways are involved in the transcriptional regulation of uPAR in cancer cells.

### 4.3. Regulation of Plasminogen Expression

The plasminogen gene maps at 6q26 and comprises 19 exons (Gene ID 5340). The PLG gene promoter contains three 3 TATA boxes at 550 to 600 bp upstream of the transcription initiation site, a TATA-like sequence (TGTAA) at position −16, and putative binding sites for several transcription factors [115].

Two regulatory sequences acting in synergism have been identified in the promoter region: the binding site of hepatocyte nuclear factor 1 (HNF 1), situated in the untranslated portion of the first exon, and the recognition site for a nuclear factor-like activator protein 3 (AP-3) at about −2.2 kb [86, 116]. These motifs are responsible for transcription and tissue specificity of the PLG gene, which is mainly expressed in the liver. Induction of the acute phase response to tissue injury, neoplastic growth, or infections causes an increased serum level of plasminogen, considered an acute phase reactant. Recent studies demonstrated that the acute phase mediator interleukin-6 (IL-6) induces hepatic expression of the PLG gene through an IL-6 responsive element (IL-6RE) located at −791 to −783 of the promoter. This stimulation appears to be mediated by the activation of the MAPKs and the transcription factor C/EBPa [86, 117, 118]. Moreover, nerve growth factor (NGF) is also able to upregulate PLG expression through the activation of two Sp1 binding sites located between nucleotides at −255 and −106 of the gene promoter [119].

### 4.4. Modulation of uPA Expression by TGF-*β*

Transcriptional activation of the uPA gene can be obtained by a number of different stimuli (e.g., phorbol esters, growth factors, etc.) which act through different signal transduction pathways, which mostly target the enhancer regions [86, 94].

TGF-*β* modulates uPA expression in different types of transformed cells: one of the first studies was performed by Keski-Oja et al. [120], showing that TGF-*β* regulates the expression of uPA in A549 human lung carcinoma. This study helped the understanding of the capacity of TGF-*β* to enhance migration and invasion of transformed cells. TGF-*β* has been demonstrated to regulate uPA expression in both tumor cells and normal cells [121–128], suggesting important roles of uPA regulation in normal cell differentiation, angiogenesis, and cell development, among other cellular functions.

**Figure 4:**
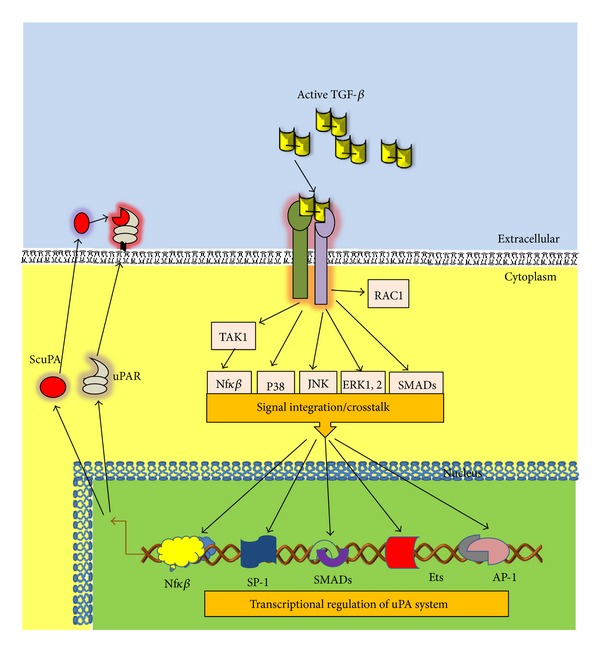
Integration of TGF-*β* signaling and transcription factor in the regulation of uPA/uPAR expression. Active TGF-*β* by binding to the receptors triggers the activation subsequent intracellular signaling, which in turn by activating transcription factors or inducing transcription factor complexes regulates uPA and uPAR expression, increasing the protein levels in cancer cells.

Although it is clear that TGF-*β* regulates uPA expression in both normal and tumor cells, the underlying mechanisms are still not well elucidated. As mentioned before, TGF-*β* activates a plethoric set of signal transduction pathways including SMAD and non-SMAD routes [7] that are involved in the regulation of uPA expression and summarized in Figure 4. We first demonstrated the implication of Ha-Ras-ERK1,2 MAPK signaling in TGF-*β*-enhanced uPA expression in transformed mouse keratinocytes [129]. Also, TGF-*β* was shown to increase uPA expression by activating the JNK pathway, implicating transcriptional regulation of uPA gene, concomitantly with the induction of EMT [130]. Furthermore, the TGF-*β* enhancement of reactive oxygen species (ROS) by Rac1-NOXs-dependant mechanism participates in NFkB-mediated uPA expression [131]. Finally, we demonstrated that SMAD3 is also required for TGF-*β* stimulation of uPA, and that the participation of SMAD3 seems to be dependent of Sky interacting protein (SKIP), since SKIP regulates SMAD3 activation and regulation of uPA expression by TGF-*β* [132]. There is divergent information about the participation of SMAD4 in the regulation of uPA expression by TGF-*β*. In breast cancer cells, SMAD4 is required for TGF-*β*-induced uPA, whereas exogenous expression of SMAD4 in colon cancer cells reduces uPA production [133, 134]. This could be explained by SMAD4 being a common SMAD for TGF-*β* and other members of the TGF-*β* superfamily such as bone morphogenetic proteins (BMPs), and its effect can also depend on the cell context [14].

TGF-*β* may induce uPAR expression [135]; however, the mechanism of this regulation has not been well studied yet. Similarly to uPA expression, a set of transcription factors involved may be regulated by TGF-*β* signaling; therefore, it is plausible to speculate that uPAR expression can in the same way be regulated by TGF-*β*, although further studies are necessary to elucidate by which mechanism.

### 4.5. Epigenetic Regulation of uPA and uPAR

The epigenome of cancer cells displays numerous alterations in comparison to the epigenome of their normal counterpart [136]. An increasing body of evidence indicates that epigenetic alterations such as modifications in DNA methylation of the CpG islands in the 5′-flanking region of genes and changes in chromatin structure by histone modification appear to play an important role in the regulation of gene transcription [86, 137]. In analogy to genetic mutation, tumors seem to accumulate higher levels of aberrant DNA methylation during tumor progression and tumorigenesis leading to inappropriate gene expression [136]. In breast cancer cells, a hypomethylation of uPA promoter has been correlated with the overexpression of uPA in high invasive MDA-MB-231 cell line, whereas a silencing of uPA expression was found to be associated with uPA promoter hypermethylation in low malignant MCF-7 cells [86, 101]. In prostate cancer cells, the increase in uPA expression has also been associated with uPA promoter hypomethylation [138].

Similarly, uPA gene transcription is subject to repression by histone deacetylation, as shown by the use of histone deacetylase (HDAC) inhibitors, such as sodium butyrate and trichostatin, which enhanced uPA expression and cancer cells invasion [139]. These observations imply that caution is required in the use of HDAC inhibitors in cancer therapies, since they might increase tumor malignance by inducing uPA expression in cancer or stromal cells [86].

Although a substantial amount of work has been done to identify the cis- and transacting factors regulating uPAR expression, the epigenetic regulation of this gene is poorly understood. It was recently found that histone variant H2A.Z is repressive for the expression of uPAR. Chromatin immunoprecipitation assays revealed that H2A.Z was enriched at previously characterized u-PAR-regulatory regions (promoter and a downstream enhancer) and that it dissociated upon activation of gene expression by PMA in an MEK1,2-ERK1,2-dependent way [140]. Understanding the molecular mechanism of epigenetic regulation of genes involved in cancer and metastasis might, ultimately, lead to the development of drugs that corrects the expression of epigenetically dysregulated genes.

Whether TGF-*β* regulates uPA/uPAR in cancer cells by epigenetic mechanism still remains unanswered. It was recently reported that the TGF-*β* receptors-SMAD2 axis is involved in the maintenance of epigenetic silencing of critical genes for the maintenance of epithelial phenotype of breast cancer cells [141]. Histone modification in cancer cells has also been under the influence of TGF-*β* signaling [142], indicating that TGF-*β* may influence uPA/uPAR expression during tumor progression by epigenetic mechanism, and surely future studies will help elucidate this remaining question.

## 5. Activation of Latent TGF-***β*** by uPA

**Figure 5:**
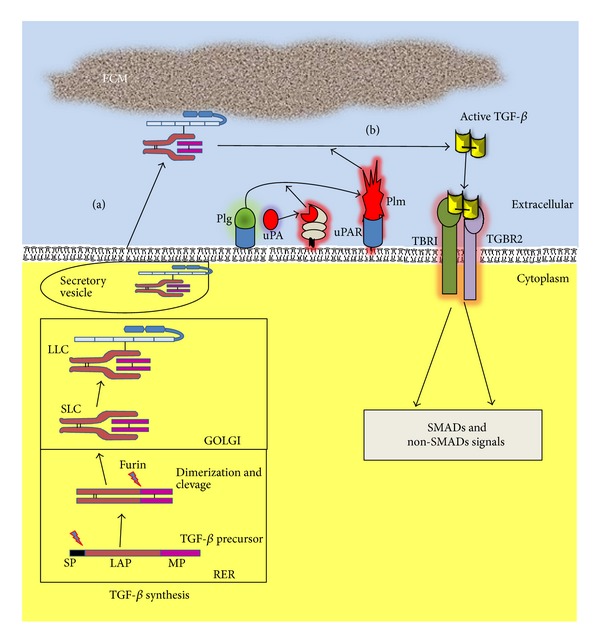
TGF-*β* processing and uPA-uPAR-dependent activation. (a) TGF-*β* is synthesized as a precursor protein. The signal peptide (SP) targets TGF-*β* precursor protein to the secretory pathway, which is cleaved during the transit through the rough endoplasmic reticulum (RER); a homodimer of protein is formed and then is cleaved by furin convertase to produce small latent complex (SLC), in which mature TGF-*β* remains noncovalent bounded to latency-associated peptide (LAP), being in that way as inactive form. Next, SLC by covalently binding to latent TGF-*β* binding protein (LTBP) produces the large latent complex (LLC); finally, LLC is secreted and stored into the extracellular matrix (ECM) for its subsequent activation. (b) uPA bounded to its uPA receptor (uPAR) activates plasminogen (Plg) to the active form plasmin (Plm); Plm can directly degrade ECM and/or may promote the activation of latent TGF-*β* by proteolytic cleavage within the N-terminal region of the LAP.

TGF-*β* is synthesized and secreted as an inactive multiprotein precursor complex consisting of a signal peptide, latency-associated peptide (LAP) domain, and mature TGF-*β* [143]. Immediately after secretion this complex is sequestered by the ECM, hence, TGF-*β* needs to be activated and released from ECM in order to exert its cellular effects [144] (Figure 5(a)).

Forming of the inactive complex begins during the transit through the rough endoplasmatic reticulum, when the first proteolytic cleavage of the precursor protein occurs, which eliminates the hydrophobic signal peptide, thus, producing a dimeric pro-TGF-*β*. The second cleavage, by furin-like convertase, which occurs in Golgi apparatus, produces the LAP and TGF-*β* mature proteins. The noncovalent bonds between them prevent the premature activation of the 25 kDa mature peptide, forming the small latent complex (SLC). The SLC is bound to a latent 125–160 kDa TGF-*β* binding protein (LTBP) via a disulphide bond giving rise to the large latent complex (LLC), which upon secretion may be covalently linked to the ECM [143–145]. The N-terminal region of LTBP is covalently cross-linked to the ECM by extracellular tissue transglutaminase. The hinge domain of LTBP is a protease-sensitive region; thus, LLC can be released from the ECM by a proteolytic cleavage [146–148]. To become bioavailable and capable of binding to its cell surface receptor, TGF-b has to be dissociated from LAP in SLC and/or LLC [144].

Extracellular activation of the latent TGF-*β* is a complex and critical process in the regulation of TGF-*β* functions *in vivo*. The interaction between TGF-*β* and LAP is not covalent and can be disrupted by both proteolytic and nonproteolytic mechanisms. Physicochemical and biological variables may participate in the regulation of TGF-*β* activation, such as heat, local acidification, exposure to reactive oxygen species (ROS), thrombospondin-1 (TSP1), integrins, and proteinases [149–153].

Among proteolytic enzymes, uPA-activated plasmin has been involved in latent TGF-*β* activation in tumor cells. Plasmin may promote the activation of latent TGF-*β* by proteolytic cleavage within the N-terminal region of the LAP (Figure 5(b)) [154]; this disrupts noncovalent bonds resulting in the releasing of bioactive TGF-*β*. In a coculture system of vascular endothelial cells and smooth muscle cells [155] or in a culture of thioglycollate-elicited macropages stimulated with LPS [156], cellular-dependent activation of latent TGF-*β* seems to involve the mannose-6-phosphate/type II insulinlike growth factor receptor (M6P/IGFII-R) and uPAR [157, 158]. One plausible mechanism is that latent TGF-*β*, bound by M6P/IGFII-R, forms a complex with uPAR, allowing the activation of TGF-*β* by local cell surface generated plasmin from plasminogen by uPA which is bound to its cell surface receptor. In addition, conversion of latent TGF-*β* to active TGF-*β* is blocked by adding anti-uPA antibodies to cocultures or by preventing uPA from interacting with its cell surface receptor [159].

Intriguingly, members of the matrix metalloproteinase (MMP) superfamily have been identified as mediators of activation of latent TGF-*β* complexes, including MMP14, MMP13 (collagenase 3), MMP9, and MMP2 [160]. Active TGF-*β* potently induces the expression of these enzymes in tumor cells. uPA may also participate in the activation of MMPs, thereby, establishing a pernicious positive autocrine regulatory loop that drives tumor progression. Conversely, the serine protease HtrA1 can negatively regulate TGF-*β* signaling by cleaving and inactivating TGF-*β* [161].

## 6. TGF-***β*** and uPA in Skin Cancer

### 6.1. Epithelial Mesenchymal Transition

The discovery that the EMT generates cells with many properties of self-renewing stem cells holds the promise of resolving a major problem in cancer biology. Many types of cancer cells leaving primary carcinomas appear to rely on the EMT program to facilitate execution of most of the steps of the invasion-metastasis cascade [162, 163].

EMT is an intricate process by which epithelial cells lose their epithelial characteristics and acquire a mesenchymal-like phenotype. During the transition, the phenotypic changes involve loss of epithelial cell-cell contacts by downregulation of junctional complex members, including claudin-1, ZO-1, and E-cadherin (CDH1), typical epithelial markers. Also, apical-basal polarity is lost, concomitantly with profound reorganization of cytoskeleton and the acquisition of a motile behavior and the final development of a fibroblastic phenotype, which is essential to increase tumor cell motility and invasive cell phenotypes [164–166]. Interestingly, as E-cadherin plays a critic role in the epithelial homeostasis, its downregulation can lead to decreased expression and/or organization of additional epithelial markers, desmosomal proteins (such as plakoglobin, desmogleins, and desmoplakins). Concomitantly, increased expression of mesenchymal markers (such as vimentin, alpha-smooth muscle actin, and fibronectin) as well as extracellular matrix remodeling enzymes (such as serine-proteinases and matrix metalloproteinases) is observed collectively with profound actin cytoskeleton reorganization [167, 168]. EMT can be a new therapeutic target for treating skin ulcer, fibrosing alopecia, and malignant cutaneous cancers, including squamous cell carcinoma and melanoma.

Although it has been demonstrated in animal tumor models that EMT occurs and promotes invasion and metastasis, the direct evidence of relevance of EMT in human cancer is still being debated [167]. The existence of cells undergoing EMT in clinical specimens has been challenged [169], probably due to the fact that EMT is a transient process, and reliable markers have been lacking [167] due to the “spatial” and “temporal” heterogeneity of EMT [170]. Cells undergoing EMT may gain metastatic potential but may constitute only a small proportion of the total population of tumor cells [171]. Therefore, identification of cancer cells undergoing EMT in clinical specimens is difficult for pathologists [172].

### 6.2. The Involvement of TGF-*β* and uPA/uPAR in EMT

Currently, TGF-*β* is recognized as a master regulator of EMT, during embryogenesis and tissue morphogenesis (type 1 EMT), wound healing and tissue fibrosis (type 2 EMT), and tumor invasion and metastasis (type 3 EMT) [173]. In cancer cells, TGF-*β* cooperates with other oncogenic SMAD-dependent or independent pathways to maintain the mesenchymal phenotype of invasive/metastatic tumor cells by regulation of TGF-*β*-induced genes and downregulation of E-cadherin expression [174, 175].

Different signaling pathways have been implicated in TGF-*β*-induced EMT: TGF-*β* induces EMT by activating SMAD complexes; SMAD4 and SMAD3 are crucial in promoting EMT [15, 164, 176, 177]; conversely, SMAD2 seems to be an inhibitor of EMT since SMAD2 ablation enhances the EMT of keratinocytes [178]. TGF-*β* has also been shown to cooperate with a plethora of signal transduction pathways to induce EMT, including Ras, Rho/Rac1, ERK1,2 MAPK, p38 MAPK, JNK MAPK, Nfkb, and Wnts [33, 131, 179]. TGF-*β* activates transcriptional factors like snail and slug to regulate the expression of epithelial or mesenchymal genes [174, 180]. Snail factors are crucial mediators of TGF-*β*-induced EMT, repressing E-cadherin transcription and activating the transcription of mesenchymal genes, such as vimentin and *α*SMA. Snail promotes collagen-I synthesis and deposition and upregulates the expression of proinflammatory interleukins IL-1, -6, and -8 [181, 182]. Cells, which have undergone EMT, may show mesenchymal stem cell features [163].

Although the ability of uPA/uPAR to promote protease activation has been studied the most, it has recently been suggested that uPA/uPAR promotes cancer progression by inducing EMT mainly by protease-independent mechanisms [183, 184]. uPA/uPAR induces EMT in cancer cells by activating several intracellular signal transduction pathways such us Ras-ERK1,2 MAPK, Rac1, and PI3K-AKT [183, 184]. uPA and uPAR have been shown to play an important role in hypoxia-induced EMT, where uPAR expression is increased and the silencing of uPA/uPAR reduces EMT [185]. Also, the uPAR signaling can induce cancer stem cells properties concomitantly with EMT in breast cancer cell line [186]. Interestingly, uPA/uPAR-induced EMT seems to be reversible suggesting strategies to control uPA/uPAR, such us blocking uPA binding to uPAR as wells as targeting intracellular signals downstream of uPAR [184, 186], which may be suitable for use in human oncotherapies.

**Figure 6:**
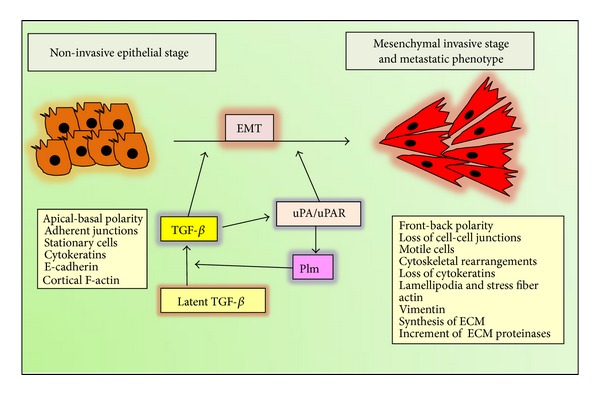
TGF-*β* and uPA system cooperation during the induction of epithelial to mesenchymal transition. Both TGF-*β* and uPA/uPAR are involved in the induction of EMT, and mutual cooperation may be operating, since TGF-*β* stimulates the expression of uPA and uPAR in cancer cells, and the enhancement of uPA levels increased the activation of plasmin, which in turn activated extracellular matrix-associated latent complex, thus, exacerbating TGF-*β*-induced EMT. Meanwhile, the increment of uPA-uPAR also stimulates EMT, and both finally may collaborate to the induction of the epithelial conversion to the mesenchymal phenotype, thus, strengthening the cancer cell invasion and metastasis.

TGF-*β* increases the expression of uPA and its binding sites on cell surface during tumor progression in the model of mouse skin carcinogenesis [187]. This increment has also been associated with TGF-*β*-induced EMT [130–132], but at the moment, it is not well known whether uPA and uPAR play a direct role in TGF-*β*-induced EMT and vice versa. It has recently been reported that bicistronic shRNA constructs targeting uPAR and cathepsin B reduced TGF-*β*1-driven invasion and survival of meningioma cells by downregulation of XIAP and pSMAD-2 expression [188], although EMT was not analyzed. It is well know that both TGF-*β* and uPA/uPAR system induce cancer-associated EMT, and it is of great importance to elucidate the interplay of both actors in the cancer scenario (Figure 6).

### 6.3. EMT in Skin Tumors

#### 6.3.1. EMT in Squamous Cell Carcinoma

In squamous cell carcinoma (SCC), cells located on the periphery of tumors are similar to epidermal stem cells, while cells exhibiting markers of terminal differentiation are usually located in the middle of the tumor [189]. Moreover, the tumor cells in the periphery display loss of surface E-cadherin and upregulation of vimentin as well as nuclear *β*-catenin staining, while cells in the tumor center remain positive for the expression of E-cadherin and cytoplasmic *β*-catenin, the typical characteristics of the epithelial phenotype [190, 191]. Although these characteristics are difficult to demonstrate in human cancers, some examples have been reported in SCC. In spindle cell squamous carcinoma, a rare variant of SCC, expressions of desmoglein-3, E-cadherin, and p120 catenin were markedly decreased and are considered as a display of EMT [192]. On the other hand, in a case of SCC mimicking atypical fibroxanthoma expression of both SNAI1 and vimentin and absence of keratin expression were observed in tumor cells [193].

In immunohistochemical staining studies of SCC, high intensity of snail and slug was associated with decreased E-cadherin staining, suggesting a correlation with the promotion of EMT [194]. Additionally, E-cadherin expression was positively correlated with *β*-catenin expression and inversely correlated with COX-2 expression in SCC cells indicating a correlation between inflammatory signals with the expression of EMT in SCC [195].

It was recently suggested that the display of EMT may contribute to the formation of cancer stem cell- (CSC-) like cells in SCC, a subset of CD29high/CD44high (where CD29 is a marker of human epidermal stem cells and CD44 as a marker to identify a subpopulation of cells with CSC properties). These findings suggested that CD29high/CD44high cells have undergone EMT from CD29low/CD44low cells and that this subpopulation may be involved in drug resistance of SCC [191].

#### 6.3.2. EMT in Malignant Melanoma

Cutaneous melanoma is an aggressive and potentially fatal form of cancer that derives from melanin-producing melanocytes in the epidermis. Melanocytes originate in the neural crest, a population of highly migratory embryonic cells [196]. Melanoma is a neoplasm of neuroectodermal origin, and because of this, melanoma cells may not undergo classic EMT-like changes. However, their ability to invade into the dermis is associated with an EMT-like phenotype characterized by changes in expression of cell-cell adhesion molecules of the cadherins family [197]. In normal skin, E-cadherin mediates contacts between melanocytes and adjacent keratinocytes. During melanoma progression, the transition from radial growth phase to invasive or vertical growth phase is characterized by decreased E-cadherin expression that results in the loss of keratinocyte-mediated growth and motility control [198]. In addition to the loss of E-cadherin, downregulation of other members of classical cadherins such as P- or H-cadherin as well as generation of a truncated secreted form of P-cadherin are frequently observed during progression of melanomas [199–201].

In melanoma cells, a regulation of Slug/SNAI2 by SPARC/osteonectin has been described, indicating that SPARC may promote EMT-associated tumor invasion by supporting AKT-dependent upregulation of SLUG [202]. Expression of slug, E-cadherin, and MITF protein in melanomas is altered during tumor progression [203].

Melanoma cells lose the capability of expressing E-cadherin, but express N-cadherin at high level *in vitro* and *in vivo*. The role of N-cadherin in melanoma metastasis is also suggested by the fact that N-cadherin promotes migration of melanoma cells over dermal fibroblasts [204]. E-cadherin expression is altered in malignant melanomas and its downregulation or absence is associated with melanoma invasion and metastasis potential. A shift from E-cadherin expression to neural N-cadherin expression in melanocytes is also detected in malignant melanomas formation [205].

A high-throughput study in melanoma identified EMT as a major determinant of metastasis; these results were confirmed in melanoma samples using tissue microarray, where a set of proteins included in the EMT group (N-cadherin, osteopontin, and SPARC/osteonectin) was significantly associated with metastasis development. These results suggest that EMT-related genes contribute to the promotion of the metastatic phenotype in cutaneous melanoma by supporting specific adhesive, invasive, and migratory properties [206].

## 7. Wound Healing

Wound healing is an evolutionally conserved, complex, multicellular process that, in skin, aims at barrier restoration. This process involves the coordinated efforts of several cell types including keratinocytes, fibroblasts, endothelial cells, macrophages, and platelets. The migration, infiltration, proliferation, and differentiation of these cells will culminate in an inflammatory response, the formation of new tissue and ultimately wound closure. This complex process is executed and regulated by an equally complex signaling network involving numerous growth factors, cytokines, and chemokines [207]. Of particular importance is the transforming growth factor-beta (TGF-*β*) family. In wound healing, TGF-*β* is important in inflammation, angiogenesis, reepithelialization, and connective tissue regeneration. It is shown to have increased expression with the onset of injury [208]. TGF-*β* facilitates the recruitment of additional inflammatory cells and augments macrophage-mediated tissue debridement [209]. It is also interesting to note that once the wound field is sterilized, TGF-*β* may be able to deactivate superoxide production from macrophages *in vitro* [210]. This helps to protect the surrounding healthy tissue and prepares the wound for granulose tissue formation.

Wound healing in skin involves three partially overlapping phases: inflammation, proliferation, and tissue remodeling. During proliferation, keratinocytes migrate and hyperproliferate at the wound edge, leading to coverage of the wound with a new epidermis, a process called reepithelialization [211].

TGF-*β*1 and TGF-*β*2 were found in the human epidermis, whereas TGF-*β*3 is distributed in the dermis, mainly in the upper dermis. TGF-*β*1 inhibits proliferation of keratinocytes, activates angiogenesis, and stimulates fibroblast proliferation and production of extracellular matrix elements. TGF-*β*2 increases protein, DNA, and collagen production [207]. TGF-*β*3 *in vivo* promotes wound healing by recruiting inflammatory cells and fibroblasts and by facilitating keratinocyte migration. TGF-*β*3 has also been shown to be a potent stimulant of neovascularization and vascular rearrangement. Furthermore, TGF-*β*3 is a potent inhibitor of DNA synthesis in human keratinocytes. These findings support the hypothesis that TGF-*β*3 may be an important stop signal for skin terminal differentiation [207, 212].

Different proteases have been implicated in the various phases of wound healing, with MMPs and serine proteases, including uPA and plasmin, being the most important [211, 213]. Plasminogen-deficient mice show severely impaired wound healing, presumably due to a diminished ability of the leading-edge keratinocytes to dissect the fibrin-rich wound matrix, and fibrin is accumulated around migrating keratinocytes. Additionally, Plg activation in skin wound is dependent on the presence of this fibrin-rich matrix [211].

The migrating leading-edge keratinocytes, during invasive phase of wound healing, express both uPA and uPAR [211, 214]. Moreover, reepithelialization of the wound in Plau-deficient mice is delayed around 50% compared to wild type mice, while no differences were observed in tissue-PA-deficient mice [215].

The physiological process, where keratinocytes detach from the epithelium and invade the wound matrix during the healing process, has been described as epithelial to mesenchymal transition with many similarities to the pathological process of tumor invasion and metastasis. This suggests that wound healing can be used as a model system for studies of cancer cell invasion [211, 216].

## 8. The Skin Carcinogenesis Model

The mouse skin model consisting of two-stage chemical carcinogenesis represents one of the best established *in vivo* models for the study of the sequential and stepwise development of tumors. In addition, this model can be used to evaluate both novel skin cancer prevention strategies and the impact of genetic background and genetic manipulation on tumor initiation, promotion, and progression [217, 218]. Mouse skin chemical carcinogenesis has provided a paradigm to study the genetic and epigenetic events which contribute to the development of squamous cell carcinomas [219].

Tumor induction in two-stage carcinogenesis involves a single subcarcinogenic dose of a carcinogen initiator, such as 7,12-dimethylbenz(a)anthracene (DMBA). This event alone does not give rise to tumors unless followed by repeated application of a tumor promoter, such as 12-O-tetradecanoylphorbol-13-acetate (TPA). This protocol gives rise to multiple benign papillomas representing clonal outgrowths of epidermal keratinocytes with initiating mutations in the HRAS1 gene, and with time, papillomas can progress to malignant SCCs [217, 220, 221].

Several studies have been performed to help the understanding of the role of TGF-*β* in the skin chemical carcinogenesis. In this carcinogenesis model, TPA rapidly induced TGF-*β* expression in keratinocytes, suggesting that endogenous TGF-*β* overexpression may contribute to TPA-mediated inflammation, as well as that it might be involved in the TPA-tumor promotion effect [222].

 When subjected to a skin chemical carcinogenesis protocol, transgenic mice overexpress TGF-*β* in the epidermis, which acts in the suprabasal layers of the epidermis exhibiting reduced papilloma formation; however, eventually as carcinogenesis progressed, TGF-*β* induced a higher rate of malignant tumors with spindle-like carcinomas cells (spSCC), thus, providing the first demonstration of TGF-*β*-induced malignant conversion *in vivo* and fitting to a well-accepted dogma, in which TGF-*β* inhibits benign tumor formation at early stages of skin carcinogenesis, but enhances malignant progression at later stages [164, 221, 223]. Similarly, studies using an inducible TGF-*β* transgene, challenged to the skin and chemical carcinogenesis protocol, showed that when TGF-*β*1 was induced early, it could suppress tumor growth, whereas when TGF-*β* was induced early in the papilloma formation stage, it actually promoted invasive tumor growth and metastasis [224].

 On the other hand, transgenic mice expressing the dominant negative mutant type II receptor of TGF-*β* in basal and follicular skin cells displayed normal tissue homeostasis by increasing both proliferation and cell apoptosis. Upon chemical carcinogenic challenge, skin cells showed a high rate of proliferation with development of a higher number of faster growing carcinomas, supporting the tumor suppressor action of TGF-*β* in the skin [164].

SMAD3 knockout mice, subjected to the two-stage chemical carcinogenesis protocol, showed a high resistance to the cancer development, indicating the importance of the intact SMAD3 signaling for the TPA-induced TGF-*β* overexpression during tumor promotion in the skin [221].

In addition, combination of oncogenic K- or HRas expression with the knockout of the type II TGF-*β* receptor in epithelial skin cells of the head and neck led to dramatic tumor growth and metastasis, associated with enhanced endogenous TGF-*β* production. The tumorigenesis was accelerated with enhanced invasiveness of the transformed keratinocytes [221]. TGF-*β* seems to be the physiological agent involved in pushing the squamous carcinoma cells to spindle carcinoma cells (SCC-SpCC) transition during mouse skin carcinogenesis, likely in cooperation with the HRAS1 oncogene [219, 225].

One of the uPA functions in epidermis is its capacity to promote keratinocyte proliferation during early stages after the mice are born, as shown in neonatal uPA−/− mice. The epidermal proliferation was affected during the first three days of mice life and normalized at day 5, which was consistent with the expression of uPA mRNA in normal mice which is high at birth and then gradually declines [226].

Consistently, the overexpression of both uPA and uPAR in the basal keratinocytes of murine skin resulted in several cutaneous alterations including a large increase in epidermis thickness with up to 24 cell layers compared to the 2-3 layers present in the wild type epidermis [50, 227]. The phenotype was due to the catalytic activity of uPA, since bitransgenic mouse overexpressing uPAR and a catalytically inactive uPA did not show epidermis hyperproliferation. In addition, upregulation and activation of MMP2 and MMP9 concomitantly with uPAR cleavage were observed. Also, it was accompanied with an increased activation of Plg, which was shown to be essential for uPA/uPAR inducing phenotype in mouse skin, as demonstrated by backcrossing the uPA/uPAR bitransgenic mice into plasminogen-deficient background, which completely recovered the normal skin phenotype [227].

In addition, TPA treatments have been shown to increase uPA levels in mouse skin. Strong signals for both uPA and PAI1 mRNA were detected earlier after treatment in the basal and suprabasal epidermal keratinocytes; later, both uPAR and PAI2 mRNAs were expressed in the epidermal layers from the suprabasal keratinocytes. In the dermis uPA mRNA was detected in fibroblast-like cells below and around skin muscle, whereas PAI1 was detected in stromal compartment above the skin muscle [228].

*In vivo*, during the induction of SCC and spSCC in the two-stage of carcinogenesis model, the direct role of uPA has not been studied. However, similarly to this protocol, a requirement of uPA during the induction of primary cutaneous melanocytic neoplasms was shown. The sequence of cellular events associated with the histological development of DMBA-induced malignant melanoma has been described [229]. Initially, small pigmented macules arise from and around an area of dorsal hyperpigmentation. These lesions progress to larger raised nevi histologically identical to human blue nevi and consist of heavily pigmented bipolar melanocytes and lightly pigmented to amelanotic spindle cells. These lesions progress to become CBN containing denser populations of spindle and epithelioid cells interspersed with melanin. Malignant melanomas ultimately appear as dermal spindle cell neoplasms frequently associated with areas of necrosis and ulceration [229, 230]. When melanocytic neoplasms were induced in uPA−/− and wild-type uPA+/+ C57BL/6, no melanomas were induced in the uPA−/− mice, which suggests that uPA contributes to malignant progression [230].

*In vitro* studies suggested the interplay between the induction of uPA by TGF-*β* and its implication in TGF-*β*-promoted tumorigenesis in late stages of metastasis diseases. In transformed keratinocytes, TGF-*β* potently induces EMT [3]. We have shown that the expression of uPA as well as uPA cell binding capacity paralleled with the increment of malignance. In immortalized keratinocytes, TGF-*β* induces temporal uPA expression, which declines to basal levels concomitantly with TGF-*β*-induced apoptosis resembling terminal keratinocyte differentiation. In cells representing SCC from stage II and III, TGF-*β* increased uPA and PAI1 and cell invasion capacity, and the cells were refractory to TGF-*β*-induced apoptosis. In spSCC expressing oncogenic HRAS1 version, cells did not respond by increasing uPA but have strongly increased PAI1 alongside with the inhibition of *in vitro* cell invasion [128, 129].

It is of great interest to delineate *in vivo* whether overexpression of TGF-*β*, during two-stage carcinogenesis protocol, may be directly involved in the increment of uPA/uPAR expression, and if together collaborates in promoting late stage of tumor progression.

It is worthy to mention the tumour suppressor role of TGF-*β* in the early steps of carcinogenesis. TGF-*β* potently inhibits epithelial cell proliferation [231], but also the tumour suppressor action of TGF-*β* can be mediated by signalling in tumour stromal fibroblasts, by inhibiting stromal uPA production, reducing local uPA production, cell motility, and uPA protection of cell apoptosis and uPA-induced angiogenesis [227], which might also contribute to TGF-*β* suppressor effects. At this time, no studies have been performed to determine the effect of TGF-*β* on stromal cells or cancer-associated fibroblast (CAFs) from tumours in the regulation uPA expression. Many questions remain to be answered, that is, what are the responses of stromal cells from different tumour stages to TGF-*β*; which factors may influence stromal uPA expression regulation by TGF-*β*. In normal gingival fibroblasts, TGF-*β* inhibits uPA expression, while in fibroblast from gingivitis areas, TGF-*β* increases uPA, and a link between inflammatory conditions to the differential TGF-*β* response has been suggested [232]. A similar mechanism could operate during tumour progression, since inflammatory response in tumour may condition cancer development [233]. Nonetheless, further, more in depth studies are necessary to elucidate the participation of the stromal compartment to the dual role of TGF-*β* in tumour progression, and on the potential differential uPA regulation by TGF-*β* during cancer development.

## 9. Skin Cancer in Humans

Skin cancer is currently the most common type of human cancer. Furthermore, it is of particular concern that its incidence is increasing at an astonishing rate. Epidemiological and molecular data strongly suggest that nonmelanoma skin cancers are associated with excessive exposure to the ultraviolet (UV) radiation in sunlight [234, 235]. The majority of human epithelial cancers (>85%) including pancreatic, colon, breast, prostate, and lung have aberrations in components of the TGF-*β* signaling pathway. A number of neoplasms originate from cutaneous epithelial cells, the most common of which are basal cell carcinoma (BCC) and squamous cell carcinoma. Interspersed among epithelial cells are pigment-producing melanocytes, which give rise to malignant melanoma (MM). Although widespread and increasing in incidence, BCC, SCC, and MM have been poorly understood at the level of molecular pathogenesis until recently [236]. Next, we will analyze the roles of TGF-b and uPA/uPAR in human skin cancer, which is summarized in Table 1.

### 9.1. Basal Cell Carcinoma

BCC is the most common cancer in fair-skinned populations. Histologically, BCCs lack precursor lesions and can be subdivided into a number of sub-types, including superficial, nodular and aggressive-growth, or morpheaform. Based on morphologic observations in tissue sections, it is believed that a substantial proportion of all BCCs may arise from hair follicle keratinocytes. Clinically, BCCs are characterized by local invasion and contiguous spread. While reports of metastatic BCC exist in the literature, it is widely recognized that BCC metastasis is an extremely rare event, in contrast to SCC (revised in [236, 237]).

Several studies have shown markedly reduced or negative expression for TGF-*β*s and SMAD proteins in BCCs compared with normal epidermis, while expression of TGF-*β* and its receptors TBRI and TGFBR2 were enhanced in the peritumoral stroma. These data indicate a potential growth inhibitory escape mechanism for BCCs by downregulating TGF-*β* in tumor cells. They also suggest a possible role for TGF-*β* signaling in stromal cells that could contribute to tumor local invasion [238–240]. Conversely, TGF-*β*, SMAD2, and SMAD3 have been shown to be overexpressed in human BCC in comparison with nonlesional skin [241, 242], suggesting a dysregulation of TGF-*β* signaling in BCC.

Based on indirect observations, TGF-*β* might also be implicated in BCC through its crosstalk with Hedgehog (Hh) signaling, which has been shown to be deregulated in BCC [237, 243]. The binding of Hh to PTCH1 receptor triggers activation of Gli family of transcription factors. Current evidence suggests that Hh pathway deregulation alone can rapidly generate BCC directly from normal keratinocytes [237]. Moreover, TGF-*β* expression may be regulated by the Hh signaling, and TGF-*β*-SMAD cascade can upregulate Gli transcription factor, indicating a putative positive crosstalk in BCC [243]. However, there is no direct experimental or clinical evidence for the collaboration of the TGF-*β* signaling with Hh pathway in BCC.

In BCC, neither uPA nor PAI1 has been overexpressed even in tumors infiltrating the deep layers of the dermis [244]. Similarly, another study [245] supports the low expression of uPA in BCC, which was accompanied with no changes in uPAR expression, but a small enhancement of PAI1 expression. Intriguingly, by using *in situ* hybridization methodology, Spiers et al. [246] have shown an increment of the uPA transcript, and the signal for uPA was elevated and pronounced in areas where the epidermis merged into invasive basal cell carcinoma in the superficial papillary dermis in some cases. Nonetheless, uPA system was shown to have low expression in BCC correlating with its failure to metastasize surrounding tissues.

### 9.2. Squamous Cell Carcinoma

 SCCs develop from benign precursor lesions as a result of a multistep process involving several genetic and epigenetic alterations that likely affect a number of distinct pathways. SCCs are thought to arise from the interfollicular epidermis, since they show characteristics of interfollicular epidermal differentiation [247]. SCC is a biologically aggressive tumor and may metastasize at frequencies reported between 1 and 12.5%. Following local invasion and tissue destruction, SCC commonly metastasizes to lymph nodes [236].

In human SCC samples, TGF-*β* was overexpressed either suprabasally or throughout the tumor epithelia, including basal proliferative cells [248] suggesting that TGF-*β* is overexpressed in human SCC similar to its mouse counterpart skin carcinogenesis model, where it has been demonstrated that TGF-*β* promotes metastasis in the late stage [223]. However, whether TGF-*β* has a tumor promoting role for the development of SCC in human skin is not well understood yet. Using HaCaT cells harboring mutant c-Ha-Ras, as a representative of early stage skin SCC in the model of tumor progression, Davies et al. [249] have overexpressed TGF-*β*1 or TGF-*β*2 which resulted in more malignant phenotypes both in organotypic cultures or tumors formed in athymic mice. Conversely, the same group [250] demonstrated that expression of a dominant-negative TGFBR2 in cells representing the later stages of tumor progression in the HaCaT model inhibited metastasis, indicating that in late stages a dysregulation of TGF-*β* signaling may be necessary. Moreover, in human clinical samples of SCC, a diminution of phospho-SMAD2 was observed in tumor samples, and in some tumors, there was evidence of a loss of pSMAD2 expression at the invasive front, which can be interpreted to in the light of that SMAD2 acts as a repressor of skin carcinogenesis, conversely to SMAD3.

In contrast with the observation in BCC, human SCC samples have shown incremented levels of uPA, uPAR, and PAI-1, found in the malignant cells [245, 251]. *In situ* hybridization studies demonstrated uPA mRNA expression in virtually all the cancer cells of the SCCs, while uPA and uPAR mRNA coexpressions were found in the adjacent sections of SCCs, in invading cancer cells [252].

*In vitro* studies demonstrated that TGF-*β* enhances uPA and PAI-1 expressions [253] as well as induces EMT in human benign and malignant keratinocytes [254]; however, it is still poorly understood if there is an interdependency between the uPA system and TGF-*β* in human SCCs, although animal and cell models support this notion.

### 9.3. Melanoma

In addition to keratinocytes, the epidermis contains a number of other cell types, among which are melanocytes. Derived from the neural crest, melanocytes synthesize the melanin pigment which provides cells of the skin with photo protection from mutagenic UV rays. Melanoma is less common than either BCC or SCC [236]. Melanomas are characterized by mutation in NRAS (20% of tumors), and BRAF in about 50% of cases, and are different subpopulations in melanomas cases [255].

In melanoma neoplasm, TGF-*β* expression is correlated with a more aggressive phenotype and increased local infiltration, suggesting that TGF-*β* may also stimulate the invasion and metastatic capacities of tumor cells to promote melanoma tumor progression [256]. TGF-*β* is overexpressed in nevi in melanomas, whereas normal melanocytes *in situ* lack TGF-*β* expression, consistent with the observation that SMAD2 pathway has been shown to be activated in both benign and malignant cutaneous melanocytic neoplasms [257, 258]. 

Melanoma cells exhibit increased resistance, proportional to tumor progression stage. Melanoma cell proliferation is only moderately inhibited by TGF-*β* in contrast to the strong antiproliferative effect exerted in normal melanocytes. In addition, a number of TGF-*β* target genes are induced by this factor in melanoma cells, in particular those involved in invasion and metastasis [256].

Increased TGF-*β*1 and TGF-*β*2 plasma levels are observed at later stages of tumor development, while no significant differences have been reported between those of healthy patients and those from patients with primary or locally invasive melanoma [259]. The TGF-*β* signal is important for the metastatic capacity of melanoma to bone, and both overexpression of SMAD7 (inhibitory SMAD) and the use of chemical inhibitor have been shown to be efficient in the inhibition of melanoma cells invasion into the bone in athymic nude mice experimental model [256, 260]. Moreover, overexpression of TGF-*β* in melanoma cells can greatly modify the tumor microenvironment, as it can activate stromal fibroblasts and induce extracellular matrix expression, such as collagen and fibronectin, which can provide an optimal microenvironment for the development of melanoma tumor progression and metastasis [256].

Additionally, it was postulated that GLI2 can mediate some TGF-*β* effects on melanoma bone metastasis. GLI2 has been identified as direct TGF-*β* target, independent from the Hedgehog signaling, in cutaneous melanoma and has been associated with the most aggressive tumors in patients with melanoma [261]. GLI2 knockdown in melanoma cells dramatically reduces their capacity to form bone metastases, and its basal expression in melanoma cells depends on autocrine TGF-*β* signaling. Moreover, GLI2 expression is associated with EMT, a critical event for the switch from an early radial growth phase to vertical growth phase of primary melanomas [261].

Melanoma, due to its tendency towards lymphogenic and hematogenous metastasis, is the most aggressive form of skin cancer. Several studies support an important role of the uPA system in this tumor type. Expression of uPA correlates with the metastatic potential of melanoma cells, and the expression of uPA and uPAR is increased in the late stage of melanomas [245]. uPAR can also act as a survival factor in melanoma, since siRNA inhibition of uPAR expression induced cell death via apoptosis. Furthermore, inhibition of uPAR reduced tumor growth in human melanoma skin reconstructs (a model that resembles the natural human skin environment *in vitro*) [262]. Similarly, targeting uPAR with phosphorothioate antisense oligonucleotides reduced cell proliferation and invasion of melanoma cells *in vitro*, as well as reduced the primary tumor mass and strongly decreased lung metastases in nude mice [263]. In addition, TGF-*β* enhances the adhesion of melanoma cells to the endothelium concomitantly with uPA-dependent activation of TGF-*β*, which may suggest a positive loop between TGF-*β* and uPA in melanoma invasion and metastasis [264]. Conversely, by using a panel of human melanoma cell lines established from different patients, TGF-*β* strongly inhibited cell migration and invasion. In these cells, TGF-*β* induced the expression of the uPA inhibitor PAI1 with the result of reduced activation of plasminogen to plasmin [265]. These results have been supported by the fact that TGF-*β* inhibits tumor growth after subcutaneous injection of B16F1 cells in syngenic mice by reducing uPA/uPAR expression as well as inducing PAI1 expression, suggesting a putative protective role of TGF-*β*1 during earliest stages of tumor progression [266]. Since melanoma cells have been shown to express high amounts of uPA, these results imply that TGF-*β* may provoke and unbalance of uPA-dependent proteolytic activity to inhibit tumor growth and metastasis.

Intriguingly, TGF-*β*, as mentioned above, was also shown to be a positive regulator of human melanoma metastasis, but the mechanisms operating in human melanoma concerning the TGF-*β* regulation of the uPA system remain unrevealed. Nonetheless, TGF-*β* and uPA system belong to a complex regulatory network of invasive behavior of melanoma tumor progression.

## 10. Concluding Remarks

There is a large number of evidence in the literature for an important role of the TGF-*β* and uPA system in the course of cancer progression and metastasis. Due to their importance in tumorigenesis, TGF-*β* and uPA system make attractive targets for cancer chemotherapies. Targeting TGF-*β* and uPA is already clinically tested in therapeutic approaches [14, 267, 268]. These strategies include small inhibitors of the enzymatic activities of uPA or TGF-*β* receptors, specific neutralizing antibodies, and peptide inhibitors such as p44 and A6 for TGF-*β* and uPA, respectively, as well as therapeutic approaches to inhibit the expression of TGF-*β* and uPAR/uPAR components at transcriptional level among others.

In this review, we attempted to reveal the uPA and TGF-*β* interplay in cancer cells with emphasis on skin malignancies. We believe that the inhibition of the amplification loop operated between TGF-*β* and uPA system in tumor cells could limit the tumor progression and metastasis impairing tumor dissemination, proliferation, and survival. We hope future clinical trials using combined therapies which target TGF-*β* and uPA system could increase the success of skin cancer treatment.

In addition, TGF-*β* and uPA induce the epithelial-mesenchymal transition, which enhances tumor cells migration and invasion and at the same time enhances the population of cancer-associated fibroblasts [269], which may open new avenues for the treatment of skin cancer. By regulating TGF-*β* and uPA, it might be possible to control the positive tumor microenvironment and cancer cells-stromal cells interaction.

Elucidating the complex interplay and roles of TGF-*β* and uPA system in cancer is critical for understanding their participation in the initiation, progression, and tumor metastasis and could eventually uncover potential combinatory therapeutic targets for future treatment of cancer in humans.

## Figures and Tables

**Table 1:** Role of  TGF-*β* and uPA in human skin cancer.

Skin cancer	TGF-*β*	uPA/uPAR
BCC	−/+	?
SCC	++	++
Melanoma	−/+	++
